# Emerging diagnostic markers and therapeutic targets in post-stroke hemorrhagic transformation and brain edema

**DOI:** 10.3389/fnmol.2023.1286351

**Published:** 2023-12-21

**Authors:** Ying Yao, Fei Liu, Zhaowen Gu, Jingyu Wang, Lintao Xu, Yue Yu, Jing Cai, Reng Ren

**Affiliations:** ^1^Department of Neuroscience Intensive Care Unit, The Second Affiliated Hospital, Zhejiang University School of Medicine, Hangzhou, Zhejiang, China; ^2^Department of Neurosurgery, The Second Affiliated Hospital, Zhejiang University School of Medicine, Hangzhou, Zhejiang, China

**Keywords:** diagnostic marker, therapeutic target, stroke, hemorrhagic transformation, edema, zonula occluden, S100β, albumin

## Abstract

Stroke is a devastating condition that can lead to significant morbidity and mortality. The aftermath of a stroke, particularly hemorrhagic transformation (HT) and brain edema, can significantly impact the prognosis of patients. Early detection and effective management of these complications are crucial for improving outcomes in stroke patients. This review highlights the emerging diagnostic markers and therapeutic targets including claudin, occludin, zonula occluden, s100β, albumin, MMP-9, MMP-2, MMP-12, IL-1β, TNF-α, IL-6, IFN-γ, TGF-β, IL-10, IL-4, IL-13, MCP-1/CCL2, CXCL2, CXCL8, CXCL12, CCL5, CX3CL1, ICAM-1, VCAM-1, P-selectin, E-selectin, PECAM-1/CD31, JAMs, HMGB1, vWF, VEGF, ROS, NAC, and AQP4. The clinical significance and implications of these biomarkers were also discussed.

## 1 Introduction

In strokes, the blood supply to part of the brain is interrupted or reduced, resulting in a loss of oxygen and nutrients to the brain (Li B. et al., 2022). Brain cells begin to die within minutes. Strokes can lead to permanent disability or death and are a medical emergency. A stroke is classified as ischemic, hemorrhagic, transient ischemic attack (TIA), or cryptogenic based on its cause (Campbell et al., 2019). Despite the availability of reperfusion therapies such as thrombolytic therapy and mechanical thrombectomy, hemorrhagic transformation (HT) and brain edema remain significant challenges in stroke management (Uniken Venema et al., 2022).

**FIGURE 1:**
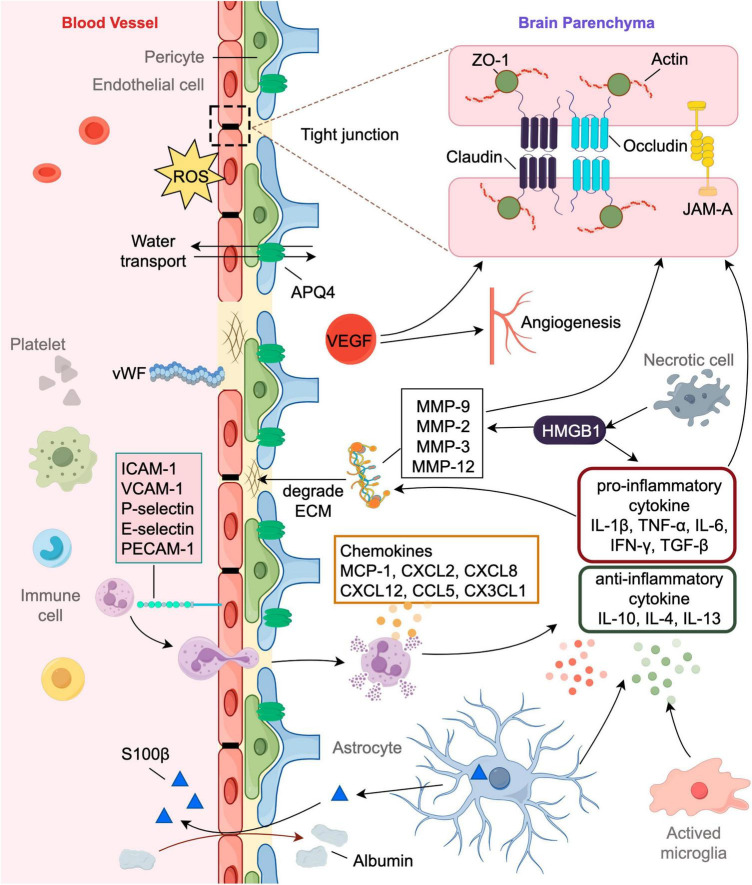
Involvement of markers in the pathophysiology of post-stroke HT and brain edema. The blood-brain barrier (BBB) consists of endothelial cells surrounded by pericytes, astrocytic end feet processes and the basement membrane, comprising extracellular matrix (ECM). Disruption of the BBB have significant implications for hemorrhagic transformation (HT) and brain edema. Tight junctions (TJs) are a key feature of the BBB, sealing the spaces between the endothelial cells and restricting the paracellular movement of solutes. TJs are complex structures comprised of various proteins like claudin, occludin, junction adhesion molecules (JAMs) and zonula occludens (ZO). Neural cells suffer from energy failure after stroke leading to inflammatory cascade and oxidative stress exacerbating BBB disruption. Pro-inflammatory cytokines can increase the expression and promote the activity of matrix metalloproteinases (MMP) which will degrade the ECM’s basal lamina. Markers like von Willebrand factor (vWF) and Vascular Endothelial Growth Factor (VEGF) might be indicative of endothelial disruption or activation, potentially predicting the risk of HT and edema. AQP4 is most abundantly located in the astrocyte foot processes that has insignificant effect on water homeostasis in the brain. It plays an important role in both the formation and resolution of post-stroke edema.

Hemorrhagic transformation represents the occurrence of bleeding within an area of the brain that has suffered an ischemic insult (Hong et al., 2021). This bleeding can happen spontaneously or be precipitated by treatments, especially those that aim to restore blood flow, like thrombolytic agents (Jickling et al., 2014). Brain edema refers to the accumulation of fluid in the brain tissue, leading to increased intracranial pressure. Following an ischemic stroke, edema can develop around the infarcted area, causing a mass effect, which can further compromise brain function and even lead to herniation, a life-threatening condition (Ji et al., 2021). Both complications are associated with poor prognosis and limited treatment options. While imaging methods like CT and MRI are commonly used to diagnose HT and brain edema, the early stages of these complications may not always be detectable (von Kummer et al., 2015). Besides, there’s a lack of universally accepted, standardized treatment protocols for managing HT and brain edema, especially in the setting of large vessel occlusions and after endovascular procedures. Current treatments for brain edema, such as osmotic therapy and decompressive craniectomy, are somewhat non-specific and come with their own set of risks (Beez et al., 2019). More targeted therapies, perhaps at the molecular or cellular level, are needed. Therefore, identifying novel diagnostic markers and therapeutic targets is essential for early detection and improved management of post-stroke HT and brain edema. This review aims to explore recent advances in the pathophysiology of post-stroke HT and brain edema, as well as the identification and potential therapeutic targets of novel diagnostic markers (Figure 1).

## 2 Blood-brain barrier (BBB) structural markers

Blood-brain barrier is a highly specialized structure in the central nervous system (CNS) that regulates the passage of substances between the blood and the brain. It plays a crucial role in maintaining a stable internal environment for neuronal function (Sweeney et al., 2019). Disruption of the BBB can have significant implications for brain health, leading to neuronal damage and various neurological conditions such as cerebral edema and brain hemorrhage (Sweeney et al., 2018). Tight junctions (TJs) are complex structures that form the primary barrier within the BBB, (Abdullahi et al., 2018). They seal adjacent endothelial cells together, ensuring a strict regulation of substance movement between the blood and the brain’s extracellular fluid. Tight junction proteins (TJPs) are crucial components of these junctions, and their integrity is essential for maintaining the BBB’s selective permeability (Berndt et al., 2019). Changes in the levels of TJPs like claudin, occluding, junction adhesion molecules (JAMs), and zonula occludens (ZO) after a stroke lead to heightened permeability of the BBB allowing for the extravasation of blood components, which might hint toward impending HT and vasogenic edema (Huang et al., 2012; Zhao et al., 2018).

Claudins are integral membrane proteins that play a pivotal role in the formation and maintenance of TJs. Among the claudin family, claudin-5 is particularly significant in the BBB (Lv et al., 2018). It forms the tight junction strands’ backbone and determines the selective permeability of the barrier (Li C. et al., 2022). While not as selectively determinant as claudins, occludin plays a role in the regulation and maintenance of TJ integrity. It is involved in the sealing of the paracellular pathway (Kim et al., 2020). ZO-1 is a cytoplasmic scaffolding protein that ensures structural stability by linking transmembrane proteins like claudins and occludin to the actin cytoskeleton, providing structural support (Gao and Shivers, 2004). This interaction stabilizes and strengthens the tight junctions, ensuring the selectivity and integrity of the BBB. JAM-A is an adhesion molecule that is part of the immunoglobulin superfamily found at tight junctions between endothelial cells (Williams et al., 1999; Babinska et al., 2020). It interacts with other TJPs like ZO-1 and plays a role in leukocyte transmigration, which can be relevant in neuroinflammatory conditions (Severson and Parkos, 2009; Sladojevic et al., 2014). Alterations in JAM-A expression or function can impact BBB integrity and may be associated with post-stroke complications like HT and brain edema (Haarmann et al., 2010; Xie et al., 2023). Following an ischemic stroke, there’s often inflammation and oxidative stress which can negatively impact the expression, structure, and function of TJPs (Yang et al., 2019). Disrupted TJs of BBB indicate an increased risk of blood products leaking into the brain tissue leading to HT, especially after reperfusion therapies or spontaneous reperfusion. With the disassembly or dysfunction of TJs, there’s an osmotic imbalance created by the abnormal influx of water and solutes from the bloodstream into the brain tissue. This results in cytotoxic and vasogenic edema, increasing intracranial pressure and exacerbating brain injury (Corrigan et al., 2016).

## 3 Matrix metalloproteinases (MMPs)

Matrix metalloproteinases are zinc-dependent proteolytic enzymes that degrade the extracellular matrix (ECM). A variety of physiological processes rely on them, including tissue remodeling, embryogenesis, and wound healing (de Almeida et al., 2022). In the context of ischemic stroke, certain MMPs are implicated in the disruption of BBB by degrading TJPs and basal lamina components directly, which will promote HT and brain edema (Rempe et al., 2016). Besides, several MMPs are reported to cleave and activate various inflammatory mediators, exacerbating post-stroke inflammation, and further contributing to brain edema.

Of the MMPs, MMP-9 (gelatinase B) has been most prominently tied to BBB disruption following ischemic stroke degrading type IV and V collagens, which are significant components of the ECM’s basal lamina as well as tight junction proteins including occludin, claudins, and ZO-1 (Yang et al., 2020). This degradation weakens the structural integrity of BBB, making it more susceptible to disruption. Hence, upregulated levels of MMP-9 in the serum or CSF after stroke have been correlated with worse clinical outcomes and increased risk of hemorrhagic transformation and vasogenic edema (Rosell et al., 2008; Barr et al., 2010; Mechtouff et al., 2020). MMP-9 can also activate microglia and attract leukocytes to the site of injury, further promoting inflammation (Qiu et al., 2023). This inflammatory cascade can exacerbate brain edema. Additionally, MMP-9 contributes to cytotoxic edema by indirectly impairing cellular ion and water balance mechanisms. MMP-2 (gelatinase A) can degrade type IV collagens. Apart from its role in ECM degradation, MMP-2 can induce endothelial cell apoptosis (Zhang S. et al., 2018). Loss of endothelial cells further weakens the BBB structure and function. It’s constitutively expressed in the brain and can be upregulated after cerebral ischemia. MMP-3 (stromelysin-1) can activate other MMPs, including MMP-9, amplifying the proteolytic effects. It also has substrate specificity for various extracellular matrix components, especially proteoglycans and fibronectin, potentially contributing to BBB breakdown (Chung et al., 2013). Its role in BBB disruption has been studied in conditions like neuroinflammation and traumatic brain injury. The pro-inflammatory cytokines, especially TNF-α and IL-1β, can upregulate MMP expression via activation of transcription factors like NF-κB and AP-1. This creates a feedback loop wherein inflammation promotes MMP activity, and MMPs further augment inflammation (Huang et al., 2022).

Given their significant role in BBB disruption, MMPs, especially MMP-9, have become therapeutic targets for interventions aiming to reduce the complications of ischemic stroke. Some of the MMP inhibitors have been studied in the context of stroke. Batimastat (BB-94) is a broad-spectrum MMP inhibitor that has shown neuroprotective effects in animal models of stroke when administered either before or immediately after the onset of ischemia (Zhou et al., 2021). Despite this, poor bioavailability and potential side effects restrict its clinical application. Naturally occurring endogenous inhibitors of MMPs including TIMP-1 and TIMP-2 have been of particular interest in stroke research (Zieliñska-Turek et al., 2022). Overexpression of TIMPs or using recombinant TIMPs has shown neuroprotection in animal models of ischemic stroke. Though primarily an antibiotic, minocycline has anti-inflammatory properties and can inhibit MMPs, especially MMP-2 and MMP-9 (Switzer et al., 2011). Several preclinical studies have shown that minocycline can reduce infarct area and improve neurological outcomes in animal ischemic stroke models. Some clinical trials have also been undertaken, suggesting potential benefits, though more extensive studies are needed (Ortiz et al., 2020). Preclinical studies have shown that SB-3CT, a more selective inhibitor targeting MMP-2 and MMP-9, can reduce infarct size and preserve BBB integrity in animal models of ischemic stroke (Cai et al., 2017). However, it’s crucial to approach MMP inhibition with caution, as these enzymes also play roles in tissue repair and remodeling during the recovery phase.

## 4 Inflammatory markers

Stroke triggers an inflammatory cascade consisting of the activation of immune cells and the secretion of inflammatory mediators, such as cytokines, chemokines, and adhesion molecules (Chen S.-J. et al., 2022). These molecules promote leukocyte infiltration and increase vascular permeability, ultimately resulting in BBB disruption, vasogenic edema, hemorrhagic transformation, and worse neurological outcomes (Candelario-Jalil et al., 2022). Neuroinflammation can also lead to a positive feedback loop, where inflammation worsens BBB disruption and BBB disruption exacerbates inflammation.

### 4.1 Cytokines

Cytokines are small secreted proteins released by cells that play pivotal roles in mediating inflammatory reactions by affecting cell interaction and communications. After a stroke, there’s a rapid increase in the production of pro-inflammatory cytokines which affect endothelial cell function and integrity of BBB (Qiu et al., 2021). Increased BBB permeability allows the extravasation of blood cells, plasma proteins, and fluid into the brain parenchyma, leading to HT and vasogenic edema. Some cytokines may influence cellular ion balance and osmotic regulation, indirectly contributing to cytotoxic edema. Certain cytokines can activate astrocytes, which can exacerbate brain edema due to their role in water regulation within the CNS (Song et al., 2021).

A pro-inflammatory cytokine, IL-1β is among the earliest to be released after ischemic injury. Once released, active IL-1β binds to the IL-1 receptor (IL-1R) on various cell types, including endothelial cells, neurons, and glial cells. It triggers a cascade of inflammatory events by the NF-κB pathway, resulting in the further upregulation of other pro-inflammatory mediators and reactive oxygen species (ROS). IL-1β has been shown to alter the expression and function of TJPs, promoting MMP activity, and inducing endothelial cell activation, contributing to the development of HT and brain edema.

Tumor necrosis factor-alpha (TNF-α) can activate neighboring astrocytes, endothelial cells, and other microglia, propagating the inflammatory response. TNF-α increases the level of adhesion molecules, such as ICAM-1 and VCAM-1, on brain endothelial cells (Tang et al., 2011). This facilitates the adhesion and migration of peripheral immune cells, like neutrophils and monocytes, into the brain parenchyma. The binding of TNF-α to TNFR1 activates several downstream pathways, including NF-κB Pathway, MAPK Pathway, and Caspase Pathway. Elevated levels of TNF-α have been associated with brain edema formation and might increase the risk of HT by affecting tight junction proteins and promoting matrix metalloproteinase (MMP) activity through its signaling cascades (Chen et al., 2019).

Interleukin-6 (IL-6) levels rise following ischemic stroke from affected neurons, astrocytes, and microglia (Zhu et al., 2022). This initial release can further be augmented by other pro-inflammatory cytokines such as IL-1β and Interferon-gamma (IFN-γ) (Philipp et al., 2018). IL-6 binding to its receptor, IL-6R, further associates with the gp130 signaling protein and then activates various intracellular signaling cascades, including the JAK/STAT, MAPK, and PI3K/Akt pathways (Heinrich et al., 2003). IL-6 can contribute to BBB disruption by promoting endothelial cells into activation as well as increasing the expression of MMPs through its intracellular signaling pathways (Aliena-Valero et al., 2021). IL-6 can also modulate TJPs and contribute to the breakdown of the BBB. IL-6 has been suggested as a potential biomarker for a higher risk of developing HT and brain edema after ischemic stroke (Leasure et al., 2021). Regarding hemorrhagic stroke, higher IL-6 levels at admission are related to worse prognosis regarding function, hemorrhage volume, and edema (Leasure et al., 2021).

Interferon-gamma is primarily produced by T cells and natural killer cells and has both pro-inflammatory and immune-modulating effects (Zhang G. et al., 2018). In the context of stroke, IFN-γ can influence the production of adhesion molecules and MMPs breaking down BBB integrity. IL-6 and IFN-γ trigger the JAK-STAT pathway, leading to the expression of genes that contribute to BBB breakdown and inflammation (Zhu et al., 2021).

Transforming Growth Factor-beta (TGF-β) has been involved in both protective and detrimental effects in the context of cerebral ischemia. In response to the ischemic insult, latent TGF-β is activated. Activated TGF-β binds to its receptors, specifically, TGF-β type I and type II receptors (TβRI and TβRII), and activates the downstream Smad proteins, specifically Smad2 and Smad3. These proteins then form complexes with Smad4, translocate to the nucleus, and regulate target gene transcription (Zhang et al., 2019). TGF-β has been shown to enhance the integrity of the BBB by promoting the expression of tight junction protein occludin (Hu et al., 2023). This action can be protective against BBB breakdown, a critical factor in the development of HT and brain edema. However, chronic TGF-β activation can lead to vascular remodeling and fibrosis, potentially affecting vascular fragility (Mickael and Graham, 2019). Some studies suggest that TGF-β may increase the risk of HT, possibly by altering vascular integrity or promoting angiogenesis (Zhang et al., 2021).

Unlike the pro-inflammatory cytokines listed above, Interleukin-10 (IL-10) is primarily anti-inflammatory. IL-10 might help dampen the inflammatory response after ischemic stroke, thereby potentially reducing the risk of HT and brain edema (Piepke et al., 2021). Interleukin-4 (IL-4) and Interleukin-13 (IL-13) are typically associated with anti-inflammatory responses (Xu et al., 2020). They might play roles in shifting the immune response after stroke toward a more reparative, less inflammatory phenotype, potentially reducing the risk of HT and edema.

### 4.2 Chemokines

Chemokines are a subset of cytokines specifically involved in directing the migration of cells, particularly immune cells. Following ischemic stroke, chemokines are released in the brain, leading to immune cell recruitment and activation which can contribute to BBB breakdown. The disruption of the BBB potentially results in HT and vasogenic edema. Some chemokines might directly affect the permeability of brain endothelial cells or influence osmotic regulation, contributing to edema formation (Marchetti et al., 2022).

Monocyte Chemoattractant Protein-1 (MCP-1/CCL2) is one of the most studied chemokines in the pathophysiology of ischemic stroke (Zhao et al., 2020). MCP-1 recruits macrophages and monocytes to the injury site (Stowe et al., 2012). MCP-1 can activate astrocytes, the major cell type involved in maintaining the brain’s extracellular environment (Zhou M. et al., 2022). Activated astrocytes can lead to cytotoxic edema (Liu J. et al., 2023). Elevated levels have been associated with exacerbated brain injury and edema (Schuette-Nuetgen et al., 2012). CXCL2 (MIP-2α) can promote the disruption of the BBB by inducing endothelial cell activation and neutrophil recruitment, potentially leading to the extravasation of blood components and water, culminating in HT and vasogenic brain edema (Chen et al., 2021). While CXCL2 primarily acts on immune cells, there is the potential for direct effects on brain cells like neurons, astrocytes, and microglia (De Paola et al., 2007; La Cognata et al., 2023). Activated astrocytes and microglia can produce inflammatory mediators that can contribute to cytotoxic edema and neuroinflammation. CXCL8 (IL-8) also attracts neutrophils and has been linked to inflammation, BBB disruption, and subsequent brain edema (Lv et al., 2019). CXCL12 (SDF-1) has implications in ischemic brain injury and related complications such as HT and brain edema, Post-ischemia, CXCL12 is upregulated in the brain, acting as a chemoattractant for various immune cells, including monocytes, natural killer cells, and T cells, especially via its primary receptor, CXCR4 (Wang et al., 2012; Wang D. et al., 2023). These cells, once in the ischemic tissue, can release proteolytic enzymes, such as MMPs, which degrade the extracellular matrix and weaken the BBB. In addition, CXCL12 overexpression in ischemic tissues has been found to enhance bone marrow-derived cells (BMCs) recruitment from peripheral blood and then to induce angiogenesis (Zemani et al., 2008; Shiba et al., 2009; Nguyen et al., 2023). This process increases the risk of HT and brain edema. Continuous administration of the CXCR4 antagonist AMD3100 for 2 weeks can inhibit CXCL12-induced angiogenesis in a mouse model of stroke (Selvaraj et al., 2017).

Unique among chemokines, fractalkine (CX3CL1) exists in both membrane-bound and soluble forms. It’s involved in recruiting monocytes and microglia. In the context of ischemic stroke, fractalkine signaling has been associated with both neuroprotective and neurotoxic effects, and its role in HT and brain edema is still being elucidated (Cipriani et al., 2011).

### 4.3 Adhesion molecules

Adhesion molecules play a pivotal role in mediating the interaction between leukocytes and the endothelial cells of BBB during inflammatory processes (Supanc et al., 2011). In response to inflammatory signals, such as TNF-α and IL-1β, some adhesion molecules are upregulated on endothelial cells. The overexpression or increased activity of these molecules can lead to enhanced leukocyte infiltration into the brain parenchyma, potentially exacerbating inflammatory damage. In the context of ischemic stroke, this can contribute to complications like HT and brain edema. Here are some of the key adhesion molecules associated with these complications.

Intercellular Adhesion Molecule-1 (ICAM-1) on the endothelial cells facilitates the binding and transmigration of leukocytes, especially neutrophils, and monocytes, into the brain tissue (Huang et al., 2022; Witsch et al., 2023). ICAM-1 can bind integrins on leukocytes, facilitating their adhesion to the endothelium (Singh et al., 2023). The process of leukocyte transmigration, facilitated by ICAM-1, releases various mediators, including MMPs, and compromises the TJs between endothelial cells, leading to increased permeability of the BBB (Kunze et al., 2015). Overexpression of ICAM-1 has been linked to increased BBB permeability, inflammation, and consequent brain edema and HT (Simundic et al., 2004; Ma et al., 2011; Zhang et al., 2020). A recent study from China showed that an elevated level of serum ICAM-1 was related to the increased risk of HT after acute ischemic stroke (Wu et al., 2018).

Vascular Cell Adhesion Molecule-1 (VCAM-1) primarily participates in the adhesion and transmigration of monocytes (Sun et al., 2020; Bai et al., 2023). VCAM-1 interacts with its integrin counter-receptors (mainly VLA-4) found on leukocytes, promoting their adhesion to the vascular wall and transmigrating into the ischemic zone subsequently (Eidson et al., 2021; Gao et al., 2021; Bai et al., 2023). Leukocyte transmigration will disrupt BBB as previously mentioned. Its upregulation post-stroke potentially exacerbates HT and edema (Li B. et al., 2023).

P-selectin and E-selectin located on the surface of activated endothelial cells and platelets, can initiate the tethering and rolling of leukocytes on the endothelium which is a critical step preceding firm adhesion and transmigration (Geissmann et al., 2003; Liu S. et al., 2023). P-selectin is stored in the Weibel-Palade bodies of endothelial cells and the α-granules of platelets (Ling et al., 2023). In the event of a stroke, P-selectin is rapidly translocated to the cell surface, where it plays a key role in the initial tethering and rolling of leukocytes along the endothelium by interacting with its ligands (like PSGL-1) on leukocytes (Yu et al., 2023). E-selectin is distinctly inducible and expressed on endothelial cells in response to inflammatory cytokines, particularly TNF-α and IL-1 (Takeda et al., 2002). Following the initial tethering and rolling mediated by P-selectin and E-selectin, leukocytes become more activated, leading to firm adhesion [via other molecules like ICAM-1 and VCAM-1 and subsequent transmigration into the tissue (Richard et al., 2015; Hatchell et al., 2023)]. Higher levels of selectins have been associated with exacerbated post-stroke inflammation, BBB disruption, and potential complications like HT and brain edema (Jin et al., 2011; Hase et al., 2012; Salas-Perdomo et al., 2018; Li et al., 2020). Recently a prospective study demonstrated that higher serum E-selectin levels were statistically significantly associated with an increased risk of malignant brain edema in ischemic stroke patients receiving endovascular thrombectomy treatment (Hu et al., 2022; Zhou et al., 2023).

Platelet-Endothelial Cell Adhesion Molecule-1 (PECAM-1/CD31) is an immunoglobulin-like cell adhesion molecule expressed on the surface of endothelial cells, platelets, neutrophils, and some subsets of lymphocytes (Arias et al., 2022; Fu et al., 2023). It facilitates the final steps of leukocyte migration through the endothelial cell junctions post-stroke (Arias et al., 2022). PECAM-1 plays a role in transmitting survival signals in endothelial cells, preventing apoptosis (Gao et al., 2003). Any imbalance in PECAM-1 signaling might affect the stability of blood vessels, making them more susceptible to hemorrhagic events (Mahooti et al., 2000; Maeda et al., 2009). Besides, being expressed on platelets, PECAM-1 is involved in platelet aggregation and adhesion (Soriano Jerez et al., 2021). Dysregulation in platelet function can contribute to either thrombotic events or impaired clot stability, both of which might impact the risk of HT. Masashi Maeda et al. found that combined treatment of rt-PA with tacrolimus reduces HT, which might be due to its effects on PECAM-1/CD31 in thrombotic ischemia stroke rat model (Maeda et al., 2009). Its role in ischemic stroke and its complications is multifaceted and requires further investigation.

### 4.4 High mobility group box 1 (HMGB1)

High mobility group box 1, a nuclear protein, stabilizes nucleosome formation and promotes transcription under physiological conditions (Lotze and Tracey, 2005; Park et al., 2006). However, upon ischemic injury, HMGB1 can be released extracellularly by necrotic cells and function as a damage-associated molecular pattern (DAMP) molecule (Qiu et al., 2008; Mi et al., 2019; Chen R. et al., 2022). As a DAMP, HMGB1 binds to multiple receptors, such as the advanced glycation end-products (RAGE) receptors and toll-like receptors (TLRs), particularly TLR2 and TLR4 (Yang et al., 2010, 2011; Kim et al., 2011). The binding of HMGB1 to these receptors initiates intracellular signaling pathways that amplify inflammation (Banks et al., 2023). For example, nuclear factor-kappa B (NF-κB) and the subsequent transcription of various pro-inflammatory genes were activated afterward. This results in the production and secretion of pro-inflammatory cytokines including TNF-α, IL-1β, and IL-6 (Xie et al., 2019; Xu et al., 2023). HMGB1 can also promote the expression and activity of MMPs, especially MMP-9, thus altering the function of TJPs and consequently increasing BBB permeability (Richard et al., 2017). Therefore, HMGB1, via its inflammatory effects, plays a role in the formation of HT and vasogenic edema.

Anti-inflammatory agents might reduce the risk of HT and brain edema. Statins or 3-hydroxy-3-methylglutaryl coenzyme A (HMG-CoA) reductase inhibitors, such as Atorvastatin and Simvastatin, have anti-inflammatory and provide antioxidant properties beyond their lipid-lowering effects. Some researchers have suggested that early statin therapy might be beneficial in acute ischemic stroke (Thomas et al., 2023). Glycyrrhizin was suggested as an adjuvant therapy for avoiding HT by inhibiting the HMGB1/TLR2 signaling pathway (Chen et al., 2020). Corticosteroids are powerful anti-inflammatory agents and have been considered for use in various neurological conditions, especially in brain tumors, primarily to counteract brain edema and inflammation (Kast et al., 2021). However, the application of corticosteroids in stroke remains a topic of debate due to potential risks and the varied outcomes observed in studies. Anakinra, an IL-1 receptor antagonist (IL-1Ra), is primarily used for rheumatoid arthritis. Animal studies have suggested its potential neuroprotective effects when administered post-stroke (Sjöström et al., 2021; Venugopal et al., 2021). Natalizumab and Fingolimod are immunomodulators, primarily used for multiple sclerosis. They’ve shown promise in preclinical models of stroke, primarily by reducing the immune cell infiltration into the brain (Elkins et al., 2017; Elkind et al., 2020; Leigh et al., 2021; Ma et al., 2021; Malone et al., 2023).

## 5 Endothelial activation/dysfunction markers

Markers like von Willebrand factor (vWF) and Vascular Endothelial Growth Factor (VEGF) might be indicative of endothelial disruption or activation, potentially predicting the risk of HT and edema.

Von Willebrand factor is a multimeric glycoprotein that is produced in endothelial cells and megakaryocytes and stored in Weibel-Palade bodies of endothelial cells and α-granules of platelets (Zhu et al., 2016). Under conditions of vascular injury, such as that seen during an ischemic stroke, vWF binds to exposed subendothelial collagen facilitating platelet adhesion and aggregation (Menih et al., 2017). Given this, elevated vWF levels might be seen as protective against hemorrhage. However, the dynamic between vWF, thrombogenesis, reperfusion injury, and inflammation can create conditions conducive to BBB disruption and, subsequently, HT (Zhu et al., 2016). Patients with higher vWF levels post-stroke are at a higher risk of HT, especially following thrombolytic therapy (Zhang et al., 2023). The increased risk might be related to the concomitant BBB disruption and microvascular occlusions. The association of vWF with BBB integrity has implications for brain edema. Increased BBB permeability can lead to vasogenic edema due to the influx of serum proteins and fluid into the brain parenchyma (Zhu et al., 2016). Additionally, the thrombogenic role of vWF might contribute to cytotoxic edema by exacerbating ischemic injury.

Vascular Endothelial Growth Factor (VEGF) is a potent angiogenic factor (Liman and Endres, 2012). In the context of ischemic stroke, VEGF has dual and somewhat contradictory effects, with implications for both protective and detrimental outcomes, especially concerning HT and brain edema. HT can be promoted by VEGF due to its effects on vascular permeability and the creation of fragile, immature vessels and more permeable in the affected region (Zhang et al., 2000; Lai et al., 2022). In addition, VEGF increases vascular permeability by affecting the endothelial cell tight and adherens junctions, notably through its influence on occludin, claudin-5, and ZO-1 (Weis and Cheresh, 2005). As mentioned, the enhanced vascular permeability resulting from VEGF activity leads to the influx of serum proteins and fluids into the brain parenchyma, contributing to vasogenic edema (Weis and Cheresh, 2005; Lai et al., 2022). This kind of edema increases intracranial pressure, which can exacerbate neurological deficits and potentially lead to life-threatening conditions like herniation. Conversely, VEGF promotes post-stroke angiogenesis by stimulating the proliferation and migration of endothelial cells, which contribute to tissue repair and regeneration by enhancing blood flow to affected areas (Zhang and Chopp, 2002; Kaya et al., 2005). Beyond its vascular effects, VEGF has direct neuroprotective actions. It supports neuronal survival, stimulates neurogenesis, and enhances the function of neural progenitor cells (Jin et al., 2000; Sun et al., 2003). The overall balance between VEGF’s neuroprotective vs. its vascular actions can influence post-stroke outcomes, especially in the acute phase.

Delayed enhancement of VEGF signaling might be beneficial during the recovery phase, where angiogenesis and neuroprotection can support tissue repair and functional recovery. However, in the acute phase, especially in patients undergoing reperfusion strategies, VEGF inhibition might be considered to reduce the risk of HT and severe edema. Overall, understanding the temporal dynamics of VEGF expression and signaling post-stroke, as well as individual patient factors, will be crucial for developing effective therapeutic strategies centered on VEGF.

## 6 Oxidative stress

Oxidative stress is a condition characterized by an imbalance between reactive oxygen species (ROS) production and antioxidant defense systems (You et al., 2023). During reperfusion following an ischemic stroke, the reintroduction of blood flow causes a paradoxical surge in ROS production, exacerbating the injury (Wu et al., 2021; Hong et al., 2023). Oxidative stress damages the BBB by directly affecting the endothelial cells, pericytes, and basal lamina as well as activating MMPs, and inflammatory pathways indirectly (Shaheryar et al., 2023, 2023; Wang S. et al., 2023). A compromised BBB increases the risk of hemorrhagic transformation and vasogenic edema. Oxidative stress can reduce the availability of nitric oxide, a vasodilator, and impair endothelial function, making vessels more susceptible to injury and hemorrhage (Chen R. et al., 2023). Besides excessive ROS can damage cellular components, including lipids, proteins, and DNA (Ratliffe et al., 2023). This can disrupt cellular ion pumps leading to cellular swelling (Lipski et al., 2006). Oxidative stress can impact astrocyte function and the regulation of AQP4, influencing edema dynamics which will be described below in detail.

Antioxidants can help in mitigating the detrimental effects of oxidative stress. Edaravone, a free radical scavenger, has emerged as a therapeutic agent for ischemic stroke, particularly in some Asian countries (Ishihara and Suzuki, 2016; Wang et al., 2017). Its ability to neutralize ROS enables it to preserve the integrity of the BBB, potentially reducing vasogenic edema and the risk of hemorrhagic transformation. N-acetylcysteine (NAC) is a versatile compound with antioxidant properties that has been investigated for its potential neuroprotective effects in ischemic stroke (Zhou Z. et al., 2022; Chamorro et al., 2023). It serves as a precursor to glutathione, one of the primary intracellular antioxidants, and plays a critical role in scavenging free radicals, reducing oxidative stress, and modulating inflammatory processes (Alkandari et al., 2023). These features make NAC an intriguing candidate for addressing complications like post-stroke hemorrhagic transformation and brain edema. Vitamin E, as a lipid-soluble antioxidant, and Vitamin C, as a water-soluble antioxidant, have been extensively researched in the context of various health conditions, including its potential neuroprotective effects in stroke (Tang et al., 2022; Palakurti et al., 2023). Alpha-lipoic acid helps regenerate other antioxidants, like vitamins E and C and is involved in energy metabolism. Coenzyme Q10 (CoQ10), also known as ubiquinone, is a naturally occurring antioxidant found in almost every cell of the body. It participates in mitochondrial electron transport and also acts as a potent free radical scavenger (Crane, 2001). Due to its antioxidant and energy-promoting effects, CoQ10 holds promise as a potential therapeutic agent after stroke (Olga Nikolaevna et al., 2020). Melatonin is a hormone predominantly secreted by the pineal gland. Apart from its well-recognized role in sleep regulation, melatonin has garnered attention for its potent antioxidant, anti-inflammatory, and neuroprotective properties (Yawoot et al., 2023). These attributes have made it an intriguing candidate for various neuroprotective strategies, including those aimed at minimizing the aftermath of ischemic stroke (Li D. et al., 2023). While antioxidants offer a promising avenue for neuroprotection after stroke, further research, especially large-scale human trials, is needed to determine their efficacy, optimal timing, and dosing.

## 7 Aquaporin-4 (AQP4)

Aquaporin-4 is the predominant water channel in the CNS that has an insignificant effect on water homeostasis in the brain (Saadoun and Papadopoulos, 2010). Specifically, AQP4 is most abundantly located in the astrocyte foot processes that ensheath blood vessels, which is a strategic location for water transport in and out of the brain parenchyma (Han et al., 2015). The polarized distribution of AQP4 underscores its role in edema dynamics (Abbott et al., 2006). In the early phases after an ischemic stroke, the shortage of oxygen and nutrients, leading to cellular energy failure, causes failure of ion pumps, resulting in an accumulation of intracellular sodium, which subsequently draws water into cells (Chen Y.-Y. et al., 2023). AQP4 facilitates this water movement into neurons and astrocytes during this phase, leading to cell swelling, a hallmark of cytotoxic edema (Manley et al., 2000). In the later phases post-stroke, the upregulation of AQP4 expression aids in the leaking of plasma proteins and water into the interstitial space of the brain by disrupting the BBB integrity, exacerbating vasogenic edema (Datta et al., 2022). While AQP4 contributes to edema formation, it also affects edema resolution. Once the initial injury settles and repair mechanisms are activated, the excess water needs to be cleared from the brain. AQP4 assists in channeling this excess water back into the vasculature or toward the cerebrospinal fluid (CSF), helping in reducing edema (Hirt et al., 2009; Zador et al., 2009). After a stroke, ionic homeostasis is disturbed, creating osmotic gradients. AQP4 helps in equilibrating these gradients by allowing water to move in response to the osmotic differences.

Given the dual role of AQP4 in both the formation and resolution of post-stroke edema, therapeutic strategies targeting AQP4 need to be carefully timed and executed. During the early phase post-stroke, inhibiting AQP4 might help reduce cytotoxic edema (Sucha et al., 2022; Gono et al., 2023). TGN-020 is a selective AQP4 inhibitor. In animal models of ischemic stroke, administration of TGN-020 has been shown to reduce brain water content, suggesting its potential efficacy in reducing cerebral edema (Sun et al., 2022). Another approach to inhibit AQP4 is through RNA interference, targeting AQP4 mRNA and preventing its translation (Zhang et al., 2022). While RNAi technology offers specificity, its delivery and stability *in vivo* can be challenging. During recovery, promoting the AQP4 function might expedite the clearance of excess water, aiding in the resolution of vasogenic edema. To date, there aren’t many specific agents known to directly activate AQP4.

## 8 Soluble molecular markers of BBB leakage

When the BBB becomes compromised, several soluble molecules can be detected at altered levels or locations.

S100β is a small calcium-binding protein predominantly found in astrocytes within the CNS (Kleindienst et al., 2007). On account of its location and the specificity of its distribution, S100β has garnered interest as a potential biomarker for BBB integrity and astrocyte-related brain injury. Under normal physiological conditions, the levels of S100β in the bloodstream are relatively low due to the restrictive nature of the BBB. When the BBB is compromised, such as during trauma, inflammation, stroke, or after certain therapeutic interventions, there’s a heightened permeability that allows S100β to leak from the brain into the bloodstream (Taylor et al., 2022). Therefore, elevated serum levels of S100β might be indicative of BBB disruption and the potential for edema. A recent study found that pre-administration with Leonuri Herba Total Alkali (LHA) could significantly reduce neurological deficit scores, infarction volume, the serum levels of S100β (Li et al., 2021).

Albumin is normally restricted from entering the brain by the BBB. By measuring the ratio of albumin in the cerebrospinal fluid (CSF) to that in the blood (often referred to as the CSF/serum albumin quotient or Qalb), clinicians can gauge the degree of BBB disruption. An *in vivo* experiment showed that albumin aggravates cerebral edema in rats with ischemic stroke by increasing BBB permeability (Li C. et al., 2022).

Recognizing and understanding these above markers allows for better diagnostic, prognostic, and therapeutic strategies.

## 9 Unresolved questions and future directions

Essentially, deeper investigations into the mechanism of these markers, elucidating how they interact with cellular components and signaling pathways, must be conducted. Developing selective inhibitors of specific markers is crucial to avoid off-target effects and potential toxicity. Besides, longitudinal studies exploring the temporal dynamics of these markers will illustrate their role across different stages after stroke, determining the optimal timing of interventions. Most importantly, conducting clinical trials to assess the utility and reliability of these markers in predicting and monitoring hemorrhagic transformation and brain edema in stroke patients is pivotal for bridging the translational gap. Research exploring individual genetic, epigenetic, and environmental factors affecting marker expression and response will facilitate the development of personalized medicine approaches for stroke patients. Moreover, investigating the synergistic effects of multi-target interventions can provide a comprehensive approach to mitigating the complex pathological processes involved in post-stroke complications. Of course, there are other yet unidentified markers of hemorrhagic transformation and brain edema post-stroke that need further study. Addressing these unresolved questions and exploring the proposed future directions will significantly advance the field, providing innovative and effective strategies for managing post-stroke hemorrhagic transformation and brain edema.

## 10 Conclusion

Post-stroke HT and brain edema are serious complications following ischemic stroke, resulting in increased morbidity and mortality rates. This review highlights the recent advancements in diagnostic markers and therapeutic targets for these complications. By better understanding the underlying mechanisms and identifying novel diagnostic tools and therapeutic strategies, we can improve patient outcomes and reduce the burden of post-stroke HT and brain edema. Rigorous clinical trials and translational studies are paramount to bring these innovations from bench to bedside.

## Author contributions

YiY: Writing – original draft. FL: Writing – original draft. ZG: Writing – review and editing. JW: Writing – review and editing. LX: Writing – review and editing. YuY: Writing – review and editing. JC: Writing – review and editing. RR: Writing – review and editing.
